# Effects of Stress Hyperglycemia on Short-Term Prognosis of Patients Without Diabetes Mellitus in Coronary Care Unit

**DOI:** 10.3389/fcvm.2021.683932

**Published:** 2021-05-19

**Authors:** Luming Zhang, Zichen Wang, Fengshuo Xu, Didi Han, Shaojin Li, Haiyan Yin, Jun Lyu

**Affiliations:** ^1^Department of Clinical Research, The First Affiliated Hospital of Jinan University, Guangzhou, China; ^2^Intensive Care Unit, The First Affiliated Hospital of Jinan University, Guangzhou, China; ^3^Department of Public Health, University of California, Irvine, Irvine, CA, United States; ^4^School of Public Health, Xi'an Jiaotong University Health Science Center, Xi'an, China; ^5^Department of Orthopaedics, The First Affiliated Hospital of Jinan University, Guangzhou, China

**Keywords:** diabetes, stress hyperglycemia, CCU, short-term prognosis, insulin resistance

## Abstract

**Background:** Diabetes mellitus (DM) has a high morbidity and mortality worldwide, and it is a risk factor for cardiovascular diseases. Non-diabetic stress hyperglycemia is common in severely ill patients, and it could affect prognosis. This study aimed to analyze the influence of different blood glucose levels on prognosis from the perspective of stress hyperglycemia by comparing them with normal blood glucose levels and those of patients with DM.

**Methods:** A retrospective study of 1,401 patients in coronary care unit (CCU) from the critical care database called Medical Information Mart for Intensive Care IV was performed. Patients were assigned to the following groups 1–4 based on their history of DM, random blood glucose, and HbA1c levels: normal blood glucose group, moderate stress hyperglycemia group, severe stress hyperglycemia group and DM group. The main outcome of this study was 30- and 90-day mortality rates. The associations between groups and outcomes were analyzed using Kaplan–Meier survival analysis, Cox proportional hazard regression model and competing risk regression model.

**Results:** A total of 1,401 patients in CCU were enrolled in this study. The Kaplan–Meier survival curve showed that group 1 had a higher survival probability than groups 3 and 4 in terms of 30- and 90-day mortalities. After controlling the potential confounders in Cox regression, groups 3 and 4 had a statistically significant higher risk of both mortalities than group 1, while no difference in mortality risk was found between groups 2 and 1. The hazard ratios [95% confidence interval (CI)] of 30- and 90-day mortality rates for group 3 were 2.77(1.39,5.54) and 2.59(1.31,5.12), respectively, while those for group 4 were 1.92(1.08,3.40) and 1.94(1.11,3.37), respectively.

**Conclusions:** Severe stress hyperglycemia (≥200 mg/dL) in patients without DM in CCU may increase the risk of short-term death, which is greater than the prognostic effect in patients with diabetes. Patients with normal blood glucose levels and moderate stress hyperglycemia (140 mg/dL ≤ RBG <200 mg/dL) had no effect on short-term outcomes in patients with CCU.

## Introduction

Diabetes mellitus (DM) has developed into one of the most common chronic diseases worldwide, with its incidence increasing from 108 million (4.7%) in 1980 to 425 million (8.5%) in 2017 and an estimated increase to 629 million in 2045 (1). It has a high mortality rate (2), thus causing a serious burden of disease. In addition, patients with DM are several times more likely to suffer from cardiovascular diseases, especially coronary heart disease, than those without DM, and the disease progresses rapidly (3, 4). Studies showed that insulin resistance (5) is closely related to the occurrence of atherosclerosis, moreover, insulin resistance and hyperinsulinemia in type 2 DM patients are closely related to coronary heart disease (6). Up to 50% of patients with DM die due to complicated cardiovascular disease (7).

Stress hyperglycemia refers to a brief increase in blood glucose during acute physiological stress in the absence of DM, and it is very common in emergency and critically ill patients (8). The American Diabetes Association consensus on inpatient hyperglycemia defines stress hyperglycemia or hospital-related hyperglycemia as any blood glucose level of >7.8 mmol/L (140 mg/dL) without evidence of prior DM (9, 10). Stress hyperglycemia is a basic survival response when the body is stimulated by strong factors, such as infection, trauma, and surgery (11). Stress state could lead to increased secretion of various metabolic hormones, such as glucagon, epinephrine, and growth hormone, in the body's neuroendocrine system; this increase could directly or indirectly antagonize insulin, resulting in insulin deficiency, insulin resistance (12), and elevated blood sugar (13). Stress could also aggravate the decompensation of insulin secretion, and the insulin receptors of peripheral effector cells are downregulated, which makes the tissues less sensitive to insulin. However, when stress hyperglycemia exceeds 11.1 mmol/L (200 mg/dL), it is associated with poor prognosis and an increased risk of death, as evidenced by an increase in hospital mortality and an increased risk of malignant events, such as chronic heart failure or cardiogenic shock in patients with cardiovascular disease (14, 15).

Diabetes is not only a high risk factor for cardiovascular disease (16), but also a strong negative factor, with adverse effects on both short - and long-term outcomes of patients in CCU (17, 18). Stress hyperglycemia has a prognostic effect on many critical diseases (19), including AMI (17). It may also have therapeutic significance. Intervention in patients with stress hyperglycemia can reduce the possibility of complication development and mortality by maintaining blood glucose (20). However, at present, there is no unified standard for the treatment of stress hyperglycemia (21, 22). In our study, a large database, Medical Information Mart for Intensive Care (MIMIC) IV, was used to analyze the influence of different blood glucose levels on prognosis from the perspective of stress hyperglycemia by comparing them with normal blood glucose levels and those of patients with DM, so as to provide reference for clinical treatment.

## Methods

### Data Source

Data were extracted from MIMIC-IV v0.4, an openly accessible critical care database (23). MIMIC-IV contained over 250,000 electronic admission records in Beth Israel Deaconess Medical Center from 2008 to 2019 (24).

All information regarding patient identification were re-encoded and all identifiable information were hidden. Thus, obtaining an informed consent from the patients was not necessary. The author completed data research training from the Collaborative Institutional Training Initiative to obtain database permissions. All data were obtained from the official website of Physionet (https://mimic.physionet.org/).

### Study Population

**Figure 1:**
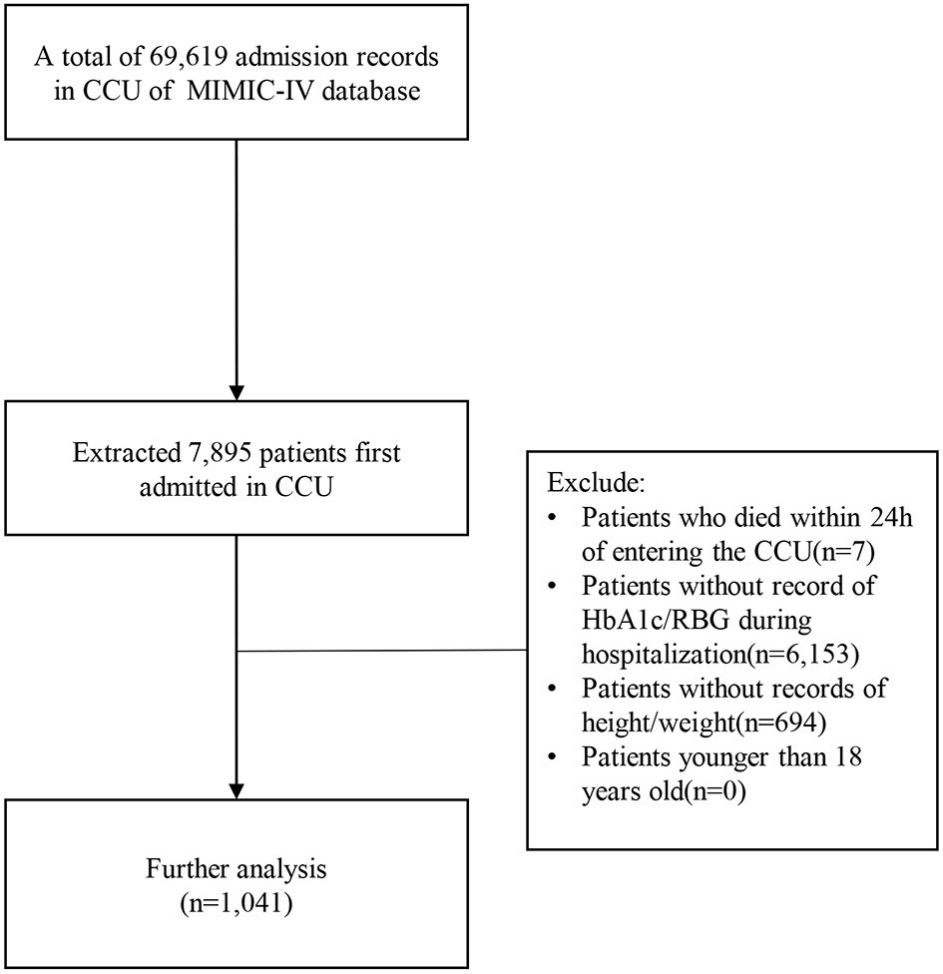
Inclusion and exclusion flowchart of the study.

Among the 69,619 CCU admission records in MIMIC-IV, 7,895 patients who were first admitted to CCU were selected. Exclusion criteria were: patients who died within 24 h of entering CCU, patients who without record of HbA1c/RBG (random blood glucose levels, patient's first blood glucose measurement after entering CCU), patients without record of height/weight, patients younger than 18 years old. In the end, a total of 1,041 patients were selected for the study (Figure 1).

### Data Extraction

Data were extracted by SQL language. The extracted variables included age, gender, BMI, vasopressor use, ventilation use, percutaneous coronary intervention (PCI), heart bypass surgery (HBS), HbA1c, RBG, acute physiology score-III (APS-III), Charlson Comorbidity Index (COMO), sepsis, ICU length of stay (LOS), hospital LOS, ICU mortality, and hospital mortality.

### Outcomes

The main outcome of this study was 30-day and 90-day mortality rates. Patients with alive discharge status and those with hospital LOS of longer than 30/90 days were defined as censors for 30-/90-day status. Otherwise, they were defined as dead. Other outcomes included ICU LOS and hospital LOS. The ICU LOS was calculated by the difference between ICU discharge time (outtime) and ICU admission time (intime). The hospital LOS was calculated by the difference between hospital discharge (dischtime) time and admission time (admittime).

### Study Cohort

All patients were divided into four groups on the basis of DM history, RBG, and HbAlc levels. Referencing the definition of stress hyperglycemia, group 1 represented the normal blood glucose group (RBG <140 mg/dL, HbAlc <6.5%, no history of DM), group 2 represented the moderate stress hyperglycemia group (140 mg/dL ≤ RBG <200 mg/dL, HbAlc <6.5%, no history of DM), group 3 represented the severe stress hyperglycemia group (RBG ≥ 200 mg/dL, HbAlc <6.5%, no history of DM), and group 4 represented the DM group (HbAlc ≥ 6.5% or history of DM).

### Statistical Analysis

The characteristics of the study population were described by median (interquartile range) for continuous variables, and as count (frequencies) for categorical data. Kruskal–Wallis test and Chi-squared test were conducted to analyze the difference in continuous and categorical variables among the four groups, respectively.

The associations between groups and the 30- and 90-day mortality rates were analyzed using Kaplan–Meier (KM) survival analysis and Cox proportional hazard regression model. The Log-rank test was conducted for non-parametric analysis to compare survival distributions between 4 groups (*p*-value < 0.05 represents a significant difference in survival between groups). Hazard ratio (HR) and 95% confidence interval (CI) were calculated via multivariable Cox regression by controlling age, gender, BMI, COMO, PCI, HBS, vasopressor use, ventilation use, APS-III, and sepsis.

We constructed a multivariable competing risk regression model to evaluate the association of patient groups and LOS in ICU/hospital (25). The included covariates are the same as the multivariate Cox regression and the time variable were ICU/hospital LOS. Since all patients will eventually be discharged, there is no censored data in this model, but patients' death in the ICU/hospital may result in a shorter LOS. Therefore, in this model, ICU and hospital LOS's failed status was defined as ICU, and hospital discharge and death are defined as a competitive risk. Therefore, for each group, HR <1 for ICU LOS represented longer ICU stay, while HR <1 for hospital LOS represented longer hospital stay compared with group 1.

Subgroup analyses were conducted for the association between groups and 30-day mortality for age (<65, ≥65), BMI (<25, 25–29.9, ≥30), PCI, myocardial infarction, congestive heart failure, APS-III ( ≤ 59, >59), and COMO ( ≤ 3, >3). The *p*-value of the overall interaction between the subgroup and the four cohorts was calculated from the Log-likelihood ratio test.

All statistical analyses were conducted on R software. Kaplan–Meier survival analysis and Cox proportional hazards regression models were performed using the “survival” package, and the competing risk regression models were analyzed using the “cmprsk” package. A two-side *p*-value < 0.05 was considered statistically significant.

## Results

Among the 1,041 patients, 337, 167, 76, and 461 were included in groups 1–4, respectively. Table 1 lists the baseline characteristics of patients. Age, gender, BMI, vasopressor use, ventilation use, COMO (details of the COMO are available in the Supplementary Material), APS-III score, 30-/90-day mortality, and ICU/hospital LOS significantly differed. Group 3 had the highest 30- and 90-day mortality rates and the longest median ICU LOS, and group 4 had the longest median hospital LOS.

**Table 1:** Baseline characteristics of the study population.

	**Group 1**	**Group 2**	**Group 3**	**Group 4**	***p*-value**
*n*	337	167	76	461	
Age (year)	63.0 (53.0, 75.0)	67.0 (55.5, 78.0)	67.5 (57.0, 77.3)	67.0 (57.0, 75.0)	0.030
Gender (%)					
Male	242 (71.8)	115 (68.9)	46 (60.5)	273 (59.2)	0.002
Female	95 (28.2)	52 (31.1)	30 (39.5)	188 (40.8)	
BMI	28.0 (24.1, 31.9)	28.1 (25.1, 31.8)	27.3 (22.9, 31.2)	29.6 (25.7, 34.6)	<0.001
Vasopressor (%)					
No	228 (67.7)	93 (55.7)	30 (39.5)	270 (58.6)	<0.001
Yes	109 (32.3)	74 (44.3)	46 (60.5)	191 (41.4)	
Ventilator (%)					
No	174 (51.6)	61 (36.5)	19 (25.0)	193 (41.9)	<0.001
Yes	163 (48.4)	106 (63.5)	57 (75.0)	268 (58.1)	
Percutaneous coronary intervention (%)					
No	261 (77.4)	134 (80.2)	66 (86.8)	382 (82.9)	0.136
Yes	76 (22.6)	33 (19.8)	10 (13.2)	79 (17.1)	
Heart bypass surgery (%)					
No	302 (89.6)	138 (82.6)	68 (89.5)	404 (87.6)	0.151
Yes	35 (10.4)	29 (17.4)	8 (10.5)	57 (12.4)	
Laboratory					
HbA1c (%)	5.6 (5.4, 5.9)	5.7 (5.4, 6.0)	5.7 (5.5, 6.0)	7.0 (6.3, 8.2)	<0.001
RBG(mg/dl)	112.0 (102.0, 125.0)	160.0 (147.0, 177.0)	238.0 (217.8, 292.3)	203.0 (152.5, 288.5)	<0.001
APS-III	36.0 (25.0, 50.0)	39.0 (29.0, 62.0)	52.0 (38.8, 73.3)	45.0 (35.0, 60.0)	<0.001
Charlson comorbidity index	2.0 (1.0, 3.0)	2.0 (1.0, 3.0)	2.0 (1.0, 4.0)	4.0 (2.0, 6.0)	<0.001
Sepsis (%)					
No	294 (87.2)	135 (80.8)	59 (77.6)	364 (79.0)	0.017
Yes	43 (12.8)	32 (19.2)	17 (22.4)	97 (21.0)	
Length of stay (Days)					
ICU	3.9 (1.6, 6.3)	4.1 (2.1, 8.4)	4.2 (2.3, 9.2)	4.1 (2.1, 7.8)	0.068
Hospital	8.0 (4.0, 14.0)	8.0 (5.0, 17.0)	8.0 (5.0, 12.0)	9.0 (5.0, 15.0)	0.046
Mortality					
30-day (%)	319 (94.7)	157 (94.0)	60 (78.9)	410 (88.9)	<0.001
No	18 (5.3)	10 (6.0)	16 (21.1)	51 (11.1)	
Yes					
90-day (%)	318 (94.4)	155 (92.8)	60 (78.9)	403 (87.4)	<0.001
No	19 (5.6)	12 (7.2)	16 (21.1)	58 (12.6)	
Yes					
ICU Death (%)					
No	322 (95.5)	156 (93.4)	60 (78.9)	416 (90.2)	<0.001
Yes	15 (4.5)	11 (6.6)	16 (21.1)	45 (9.8)	
Hospitalized Death (%)					
No	318 (94.4)	155 (92.8)	60 (78.9)	403 (87.4)	<0.001
Yes	19 (5.6)	12 (7.2)	16 (21.1)	58 (12.6)	

*Group 1 represented the normal blood glucose group, group 2 represented the moderate stress group, group 3 represented the severe stress hyperglycemia group, and group 4 represented the diabetes group*.

*Charlson Comorbidity Index was calculated by Myocardial Infarct, Congestive Heart Failure, Peripheral Vascular Disease, Cerebrovascular Disease, Dementia, Chronic Pulmonary Disease, Rheumatic Disease, Peptic Ulcer Disease, Mild Liver Disease, Diabetes(uncomplicated), Diabetes(complicated), Paraplegia, Renal Disease, Malignant Cancer, Severe Liver Disease, Metastatic Solid Tumor, and Aids*.

**Figure 2:**
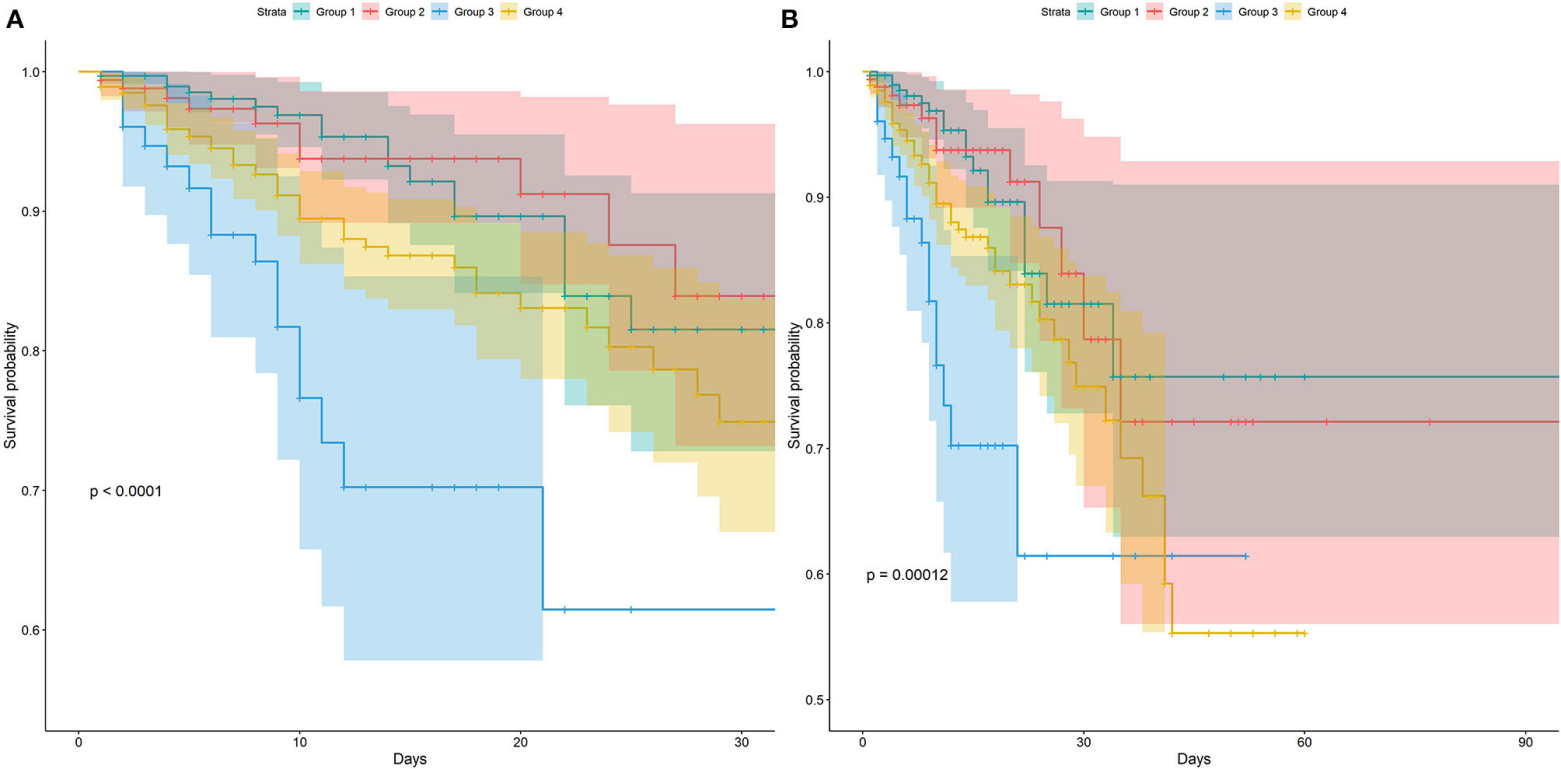
Kaplan-Meier survival curves between groups. *P*-value calculated by Log-rank test = 0.012 showed Group 3 had lower survival probability. **(A)** Represented the survival probability in 30-day. **(B)** Represented the survival probability in 90-day.

In this study, 30-day mortality is 9.1% and 90-day mortality is 10.0%. The Kaplan–Meier survival curve showed no difference in survival probability between groups 1 and 2, while group 1 had a higher survival probability than groups 3 and 4 in both mortalities (Figure 2). After controlling the potential confounders in Cox regression, group 3 had a statistically significant higher risk of 30- and 90-day mortalities than group 1, while no difference in mortality risk was found between groups 2 and 1. The HRs (95% CI) of 30- and 90-day mortalities for group 3 were 2.77 (1.39, 5.54) and 2.59 (1.31, 5.12), respectively. This finding demonstrated that compared with group 1, group 3 had 2.77 and 2.59 times of risk for 30- and 90-day mortalities, respectively. Compared to group 1, the 4 four had significantly 92 and 94% increased risk of 30- and 90-day mortality. The multivariate Cox regression results revealed that compared with the normal blood sugar group, there was no difference in the 30- and 90-day survival of the moderate stress hyperglycemia group while the death risk of severe stress hyperglycemia group and DM group was significantly increased. Besides, the severe stress hyperglycemia group presented the worst prognosis (Table 2).

**Table 2:** HRs (95% CIs) for all-cause mortality and length of stay across groups.

	**Group 1**	**Group 2**		**Group 3**		**Group 4**	
		**HR(95%CI)**	***p*-value**	**HR(95%CI)**	***p*-value**	**HR(95%CI)**	***p*-value**
30-day mortality							
Unadjusted	Reference	0.98(0.45,2.13)	0.969	4.04(2.06,7.93)	<0.001	1.87(1.09.3.20)	0.023
Adjusted	Reference	0.85(0.39,1.85)	0.673	2.77(1.39,5.54)	0.004	1.92(1.08,3.40)	0.027
90-day mortality							
Unadjusted	Reference	1.06(0.51,2.18)	0.882	3.79(1.95,7.38)	<0.001	1.95(1.16,3.28)	0.012
Adjusted	Reference	0.98(0.43,1.85)	0.752	2.59(1.31,5.12)	0.006	1.94(1.11,3.37)	0.020
ICU length of stay							
Unadjusted time to discharge	Reference	0.83(0.69,1.00)	0.044	0.56(0.42,0.74)	<0.001	0.79(0.68,0.91)	0.001
Adjusted time to discharge	Reference	1.10(0.91,1.34)	0.330	0.90(0.63.1.72)	0.540	1.08(0.90,1.28)	0.420
Hospital length of stay							
Unadjusted time to discharge	Reference	0.86(0.71,1.02)	0.090	0.61(0.46,0.80)	<0.001	0.74(0.64,0.85)	<0.001
Adjusted time to discharge	Reference	1.05(0.85,1.30)	0.650	1.04(0.79,1.36)	0.800	1.01(0.86,1.19)	0.910

*Cox proportional hazards regression models were used to calculate hazard ratios (HRs) with 95% confidence intervals (CIs)*.

*Model 1 was unadjusted*.

*Model 2 was adjusted for age, gender, BMI, COMO, PCI, HBS, vasopressor use, ventilation use, APS-III, and sepsis*.

**Figure 3:**
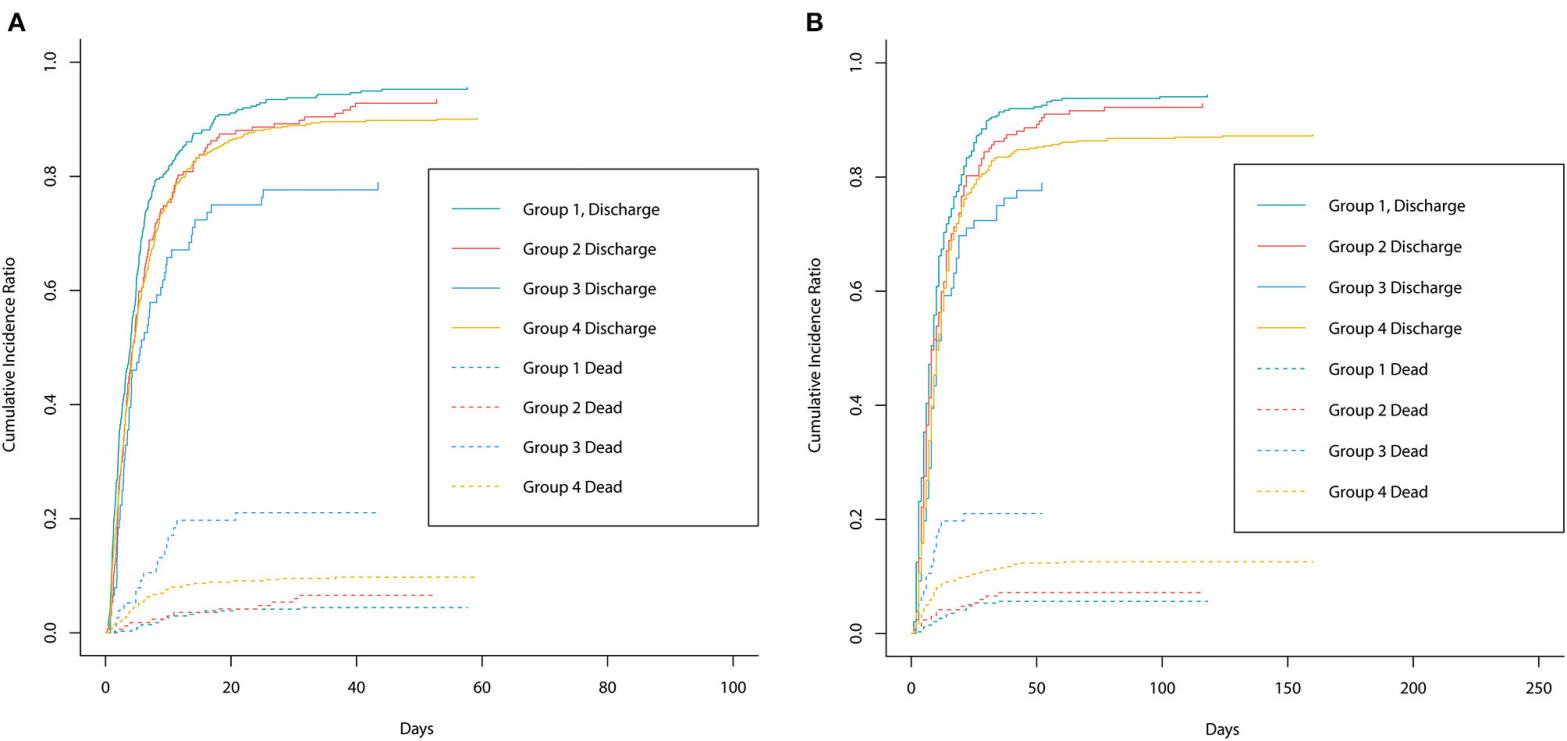
The cumulative incidence curve between groups for ICU discharge and hospital discharge. The solid line represented the cumulative incidence ratio (CIR) of alive discharged patients while the dashed line represented the CIR of dead discharged patients (competing for risk). **(A)** Represented the cumulative incidence curve for ICU discharge between groups. **(B)** Represented the cumulative incidence curve for hospital discharge between groups.

Figure 3 shows the cumulative incidence curve between groups for ICU discharge and hospital discharge. The solid line represented the cumulative incidence ratio (CIR) of alive discharged patients, while the dashed line represented the CIR of dead discharged patients (competing for risk). Figure 3A shows that group 1 had the highest increasing rate of CIR for ICU discharge, which demonstrated that groups 2–4 had longer ICU LOS. Figure 3B shows a trend similar to that in Figure 3A, indicating that groups 2–4 had longer hospital LOS. The *p*-value of univariable Fine–Gray test results for ICU LOS in groups 2–4 was <0.05 and the HR was <1, thus revealing that groups 2–4 had significantly longer ICU LOS than group 1. For hospital LOS, single-factor regression results also showed that groups 3 and 4 had significantly longer hospital LOS. However, after adjusting for confounders, the difference in ICU/hospital LOS between groups was not statistically significant (Table 2) which indicated that the effect of stress hyperglycemia and DM on the prolongation of LOS was not significant. Therefore, stress hyperglycemia and DM may have stronger impacts on the acute prognosis of CCU patients, and other variables eventually caused the longer LOS presented in univariate analysis (e.g., BMI and comorbidities).

In the subgroup analysis (Table 3), although the HR (95%CI) and the corresponding *p*-value of different groups are different. However, the *p*-values of the interaction test results are all >0.05, which indicates that other factors have no significant impact on the 30-, 90-day mortality risk of stress hyperglycemia and DM.

**Table 3:** Subgroup analysis of the associations between all-cause mortality and length of stay.

	**Group 1**	**Group 2**		**Group 3**		**Group 4**		
		**HR(95%CI)**	***p*-value**	**HR(95%CI)**	***p*-value**	**HR(95%CI)**	***p*-value**	***p*-interaction**
Age								0.202
<65 (*n* = 493)	Reference	0.13(0.02,1.04)	0.055	1.32(0.40,4.37)	0.652	0.78(0.30,2.07)	0.622	
≥65 (*n* = 548)	Reference	1.83(0.73,4.58)	0.198	3.97(1.62,9.72)	0.003^*^	3.26(1.53,6.93)	0.002^*^	
BMI								0.223
<25 (*n* = 264)	Reference	0.95(0.65,3.61)	0.941	4.71(1.67,13.30)	0.003^*^	2.19(0.90,5.31)	0.080	
25–29.9 (*n* = 358)	Reference	0.52(0.12,2.16)	0.367	1.12(0.36,3.56)	0.391	1.12(0.36,3.55)	0.839	
≥30 (*n* = 418)	Reference	1.12(0.21,5.93)	0.895	2.48(0.45,13.67)	0.296	2.54(0.72,8.92)	0.147	
Percutaneous coronary intervention								0.115
No (*n* = 843)	Reference	0.56(0.23,1.37)	0.203	2.94(1.40,6.16)	0.004^*^	1.60(0.88,2.90)	0.121	
Yes (*n* = 198)	Reference	7.98(0.57,112.61)	0.124	2.37(0.16,34.89)	0.531	8.90(0.49,162.03)	0.149	
Myocardial infarct								0.199
No (*n* = 495)	Reference	0.29(0.06,1.32)	0.110	2.24(0.70,7.15)	0.175	1.64(0.72,3.72)	0.242	
Yes (*n* = 546)	Reference	1.39(0.44,4.36)	0.570	3.38(1.15,9.93)	0.026	2.42(0.91,6.40)	0.076^*^	
Congestive heart failure								0.399
No (*n* = 443)	Reference	1.01(0.28,3.72)	0.985	2.63(0.85,8.14)	0.094	1.70(0.61,4.72)	0.312	
Yes (*n* = 598)	Reference	0.89(0.32,2.43)	0.817	3.28(1.34,8.02)	0.009^*^	2.11(1.02,4.34)	0.043^*^	
APS-III								0.202
≤ 59 (*n* = 788)	Reference	2.06(0.41,10.33)	0.378	9.62(2.35,39.49)	0.002^*^	3.61(0.99,13.16)	0.051	
>59 (*n* = 253)	Reference	0.64(0.25,1.61)	0.344	1.62(0.71,3.67)	0.252	1.46(0.75,2.88)	0.269	
Charlson comorbidity index								0.401
≤ 3 (*n* = 668)	Reference	1.20(0.46,3.14)	0.710	3.14(1.28,7.69)	0.013^*^	2.00(0.89,4.51)	0.094	
>3 (*n* = 373)	Reference	0.54(0.14,2.14)	0.384	2.61(0.85,8.04)	0.094	1.93(0.83,4.49)	0.128	

*Confounders adjustment were performed as in Model 2 (Table 2). Cox proportional hazards regression models were used to calculate hazard ratios (HRs) with 95%*.

* *p < 0.05*.

## Discussion

Considering blood glucose levels are closely associated with cardiovascular disease, 1,401 patients in CCU were selected from a large critical care medical database and adjusted for a number of potential confounders, including APS-III score and COMO. Our study showed that there was no difference in 30-day and 90-day survival in the moderate stress-hyperglycemia group compared with the normal blood glucose group, while the risk of death was significantly increased in the severe stress hyperglycemia group and the DM group. We will explain them one by one, the hyperglycemic response to stress is part of the adaptive metabolic response to severe diseases, such as trauma and infection. It is involved in neuroendocrine and immune pathways that lead to the development of insulin resistance and liver glucose production through gluconeogenesis and glycogen breakdown (26). Stress hyperglycemia forms a new glucose balance and produces a grape concentration gradient in critically ill patients, which ensures the body to absorb glucose to the maximum extent in the case of ischemia, blockage, and other microvascular blood flow obstruction. This finding is similar to that in previous research (27), which revealed that mild-to-moderate stress hyperglycemia has a protective effect in severely ill patients.

The current researches on severe stress hyperglycemia are still controversial, For example, one study (28) have shown that early post-injury blood glucose >200 mg/dL was not associated with increased infection rates and mortality in traumatic population, while another study showed that (29) that maintaining an average blood glucose level below 200 mg/dL after cardiac surgery could reduce the incidence of deep wound infection in patients with DM. Other studies have indicated that in people who don't have diabetes, short-term hyperglycemia was also associated with increased mortality after trauma and hip fracture (30, 31). Our research shows that, compared with normal blood glucose group, severe stress hyperglycemia group had 2.77 and 2.59 times of risk for 30- and 90-day mortalities, while diabetes group had 1.92 and 1.94 times of risk for both mortalities, respectively. It can be seen that, stress hyperglycemia in patients without DM causes more damage to the body than the influence of DM on the body. Research (32) has revealed that short-term hyperglycemia could reduce immunity through non-enzymatic glycosylation of circulating immunoglobulin. In addition, oxidative stress response, insulin resistance, and large amounts of catecholamine production could increase the level of free fatty acids in the body. Excessive levels of free fatty acids have toxic effects on infarcted and ischemic myocardium (33), and severe stress hyperglycemia can promote the development of patients with cardiovascular diseases (34), having a bad influence on patients in CCU. For DM patients, long-term disorder of carbohydrate, fat, and protein metabolism could cause multi-system damage, leading to chronic progressive pathological changes; functional decline; and failure of nerve, heart, blood vessels, and other tissues and organs, and they could be easily complicated with various types of infections (35). In addition, insulin resistance in diabetic patients could affect the function of vascular endothelial cells, thereby reducing the availability of nitric oxide, resulting in the increase in angiotensin and affecting blood pressure in patients in CCU (36).

Most patients in CCU are complicated with DM or insulin resistance, which affects their prognosis. Although many scholars have conducted studies on the optimal range of blood glucose control in critically ill patients, no unified standard is available as of yet (37). The present study showed that patients without DM with excessive stress blood glucose (≥200 mg/dL) had a higher risk of short-term death than patients with DM. In CCU, strengthening the control of patients' blood glucose management, developing appropriate treatment plan, improving the quality of life of patients, and reducing fatality rate are important.

This study has some limitations. First, the blood glucose level of the patients was collected for the first time after entering CCU, and whether the patients had taken blood glucose intervention measures, such as the use of insulin or hormone drugs, before entering CCU was not evaluated. Second, the types of DM were not distinguished. Finally, this study is a single-center retrospective study.

## Conclusions

Severe stress hyperglycemia (≥200 mg/dL) in patients without DM in CCU may increase the risk of short-term death, which is greater than the prognostic effect in patients with diabetes. Patients with normal blood glucose levels and moderate stress hyperglycemia (140 mg/dL ≤ RBG <200 mg/dL) had no effect on short-term outcomes in patients with CCU.

## Data Availability Statement

The datasets presented in this study can be found in online repositories. The names of the repository/repositories and accession number(s) can be found below: The data were available on the MIMIC-IV website at https://mimic.physionet.org/, https://doi.org/10.13026/a3wn-hq05.

## Author Contributions

LZ created the study protocol, performed the statistical analyses, and wrote the first manuscript draft. ZW conceived the study and critically revised the manuscript. FX assisted with the study design and performed data collection. DH assisted with data collection and manuscript editing. SL assisted the analysis and explain of statistical methods. HY assisted with manuscript revision and data confirmation. JL contributed to data interpretation and manuscript revision. All authors read and approved the final manuscript.

## Conflict of Interest

The authors declare that the research was conducted in the absence of any commercial or financial relationships that could be construed as a potential conflict of interest.
