# Supplementary material for: Effects of Stress Hyperglycemia on Short-Term Prognosis of Patients Without Diabetes Mellitus in Coronary Care Unit
Source: Front Cardiovasc Med. 2021 May 19;8:683932. doi: 10.3389/fcvm.2021.683932 (PMC8169960; doi:10.3389/fcvm.2021.683932)
Supplement: Supplementary file 1 [file Table_1.DOCX]

TableE1.Complete List of Charlson Comorbidity.

| Comorbidities | Group1 | Group2 | Group3 | Group4 | p-value |
| --- | --- | --- | --- | --- | --- |
| Myocardial Infarct (%) |  |  |  |  |  |
| No | 180 (53.4) | 72 (43.1) | 38 (50.0) | 205 (44.5) | 0.048 |
| Yes | 157 (46.6) | 95 (56.9) | 38 (50.0) | 256 (55.5) |  |
| Congestive Heart Failure (%) |  |  |  |  |  |
| No | 176 (52.2) | 73 (43.7) | 34 (44.7) | 160 (34.7) | <0.001 |
| Yes | 161 (47.8) | 94 (56.3) | 42 (55.3) | 301 (65.3) |  |
| Peripheral Vascular Disease (%) |  |  |  |  |  |
| No | 296 (87.8) | 152 (91.0) | 63 (82.9) | 388 (84.2) | 0.095 |
| Yes | 41 (12.2) | 15 (9.0) | 13 (17.1) | 73 (15.8) |  |
| Cerebrovascular Disease (%) |  |  |  |  |  |
| No | 295 (87.5) | 157 (94.0) | 67 (88.2) | 396 (85.9) | 0.054 |
| Yes | 42 (12.5) | 10 (6.0) | 9 (11.8) | 65 (14.1) |  |
| Dementia (%) |  |  |  |  |  |
| No | 335 (99.4) | 166 (99.4) | 76 (100.0) | 455 (98.7) | 0.549 |
| Yes | 2 (0.6) | 1 (0.6) | 0 (0.0) | 6 (1.3) |  |
| Chronic Pulmonary Disease (%) |  |  |  |  |  |
| No | 269 (79.8) | 130 (77.8) | 53 (69.7) | 326 (70.7) | 0.015 |
| Yes | 68 (20.2) | 37 (22.2) | 23 (30.3) | 135 (29.3) |  |
| Rheumatic Disease (%) |  |  |  |  |  |
| No | 332 (98.5) | 158 (94.6) | 72 (94.7) | 439 (95.2) | 0.054 |
| Yes | 5 (1.5) | 9 (5.4) | 4 (5.3) | 22 (4.8) |  |
| Peptic Ulcer Disease (%) |  |  |  |  |  |
| No | 335 (99.4) | 166 (99.4) | 74 (97.4) | 455 (98.7) | 0.377 |
| Yes | 2 (0.6) | 1 (0.6) | 2 (2.6) | 6 (1.3) |  |
| Mild Liver Disease (%) |  |  |  |  |  |
| No | 308 (91.4) | 152 (91.0) | 67 (88.2) | 434 (94.1) | 0.185 |
| Yes | 29 (8.6) | 15 (9.0) | 9 (11.8) | 27 (5.9) |  |
| Diabetes (uncomplicated) (%) |  |  |  |  |  |
| No | 337 (100.0) | 167 (100.0) | 76 (100.0) | 160 (34.7) | <0.001 |
| Yes | 0 (0.0) | 0 (0.0) | 0 (0.0) | 301 (65.3) |  |
| Diabetes (complicated) (%) (%) |  |  |  |  |  |
| No | 337 (100.0) | 167 (100.0) | 76 (100.0) | 329 (71.4) | <0.001 |
| Yes | 0 (0.0) | 0 (0.0) | 0 (0.0) | 132 (28.6) |  |
| Paraplegia (%) |  |  |  |  |  |
| No | 326 (96.7) | 164 (98.2) | 72 (94.7) | 445 (96.5) | 0.539 |
| Yes | 11 (3.3) | 3 (1.8) | 4 (5.3) | 16 (3.5) |  |
| Renal Disease (%) |  |  |  |  |  |
| No | 284 (84.3) | 138 (82.6) | 62 (81.6) | 293 (63.6) | <0.001 |
| Yes | 53 (15.7) | 29 (17.4) | 14 (18.4) | 168 (36.4) |  |
| Malignant Cancer (%) |  |  |  |  |  |
| No | 326 (96.7) | 159 (95.2) | 76 (100.0) | 448 (97.2) | 0.240 |
| Yes | 11 (3.3) | 8 (4.8) | 0 (0.0) | 13 (2.8) |  |
| Severe Liver Disease (%) |  |  |  |  |  |
| No | 335 (99.4) | 160 (95.8) | 74 (97.4) | 455 (98.7) | 0.020 |
| Yes | 2 (0.6) | 7 (4.2) | 2 (2.6) | 6 (1.3) |  |
| Metastatic Solid Tumor (%) |  |  |  |  |  |
| No | 329 (97.6) | 165 (98.8) | 76 (100.0) | 457 (99.1) | 0.208 |
| Yes | 8 (2.4) | 2 (1.2) | 0 (0.0) | 4 (0.9) |  |
| Aids (%) |  |  |  |  |  |
| No | 336 (99.7) | 167 (100.0) | 76 (100.0) | 461 (100.0) | 0.554 |
| Yes | 1 (0.3) | 0 (0.0) | 0 (0.0) | 0 (0.0) |  |
